# The amelioration of ovarian dysfunction by hesperidin in malathion-treated mice through the overexpression of PCNA and FSHR proteins

**DOI:** 10.1016/j.heliyon.2023.e22484

**Published:** 2023-11-22

**Authors:** Mahnaz Zarein, Asghar Zarban, Hamed Shoorei, Mehdi Gharekhani, Mohammadmehdi Hassanzadeh-Taheri

**Affiliations:** a Department of Anatomical Sciences, Faculty of Medicine, Birjand University of Medical Sciences, Birjand, Iran; b Clinical Biochemistry Department, Faculty of Medicine, Birjand University of Medical Sciences, Birjand, Iran; c Cellular and Molecular Research Center, Birjand University of Medical Sciences, Birjand, Iran; d Clinical Research Development Unit of Tabriz Valiasr Hospital, Tabriz University of Medical Sciences, Tabriz, Iran

**Keywords:** Malathion, Hesperidin, PCNA, FSHR, Ovary

## Abstract

**Objective:**

Malathion (MAL), a pesticide used for decades, is a highly toxic substance. Several studies have documented the negative effects of such agents on reproductive organ physiology, but the precise mechanism of action in the induction of ovarian dysfunction remains unclear. Therefore, the purpose of this research was to examine the effects of the antioxidant hesperidin (HES) on ovarian damage and toxicity caused by malathion.

**Materials and methods:**

In this experiment, forty adult female bulb/c mice weighing 27–30 g were categorized into four groups, namely hesperidin (20 mg/kg, i.p.), malathion (3 mg/kg, i.p.), malathion + hesperidin, and control groups. Following a period of 35 consecutive days of treatment, mice were euthanized, and their ovarian tissues were gathered for the purposes of histopathological analysis by H&E staining, immunohistochemical assessment via proliferating cell nuclear antigen (PCNA) and follicle-stimulating hormone receptor (FSHR) immunostaining, and biochemical evaluation via measuring the levels of malondialdehyde (MDA), superoxide dismutase (SOD), catalase (CAT), tumor necrosis factor-alpha (TNF-α), and interleukin-1 beta (IL-1β). In addition, serum samples were collected from the blood of mice to perform hormonal analyses, especially 17β-estradiol (E2), progesterone (P4), luteinizing hormone (LH), and follicle-stimulating hormone (FSH).

**Results:**

The results demonstrated that MAL exposure resulted in the development of abnormalities in the architecture and structure of ovaries. Also, the treatment of mice with MAL led to declined follicular counts at all three stages, namely, primary, secondary, and tertiary, reduced serum levels of sex hormones, decreased immunoreactivity of FSHR and PCNA, and diminished activity of CAT and SOD enzymes. In contrast, it caused an increase in MDA, IL-1β, and TNF-α, as well as the count of atretic follicles. Nonetheless, it was observed that HES exhibited the ability to ameliorate the deleterious impacts of malathion across all the aforementioned parameters.

**Conclusion:**

Treatment with HES via upregulating the protein expression of PCNA and FSHR and activating antioxidant defense was able to ameliorate the adverse effects of MAL on ovarian tissues.

## 1 Introduction

**Fig. 1:**
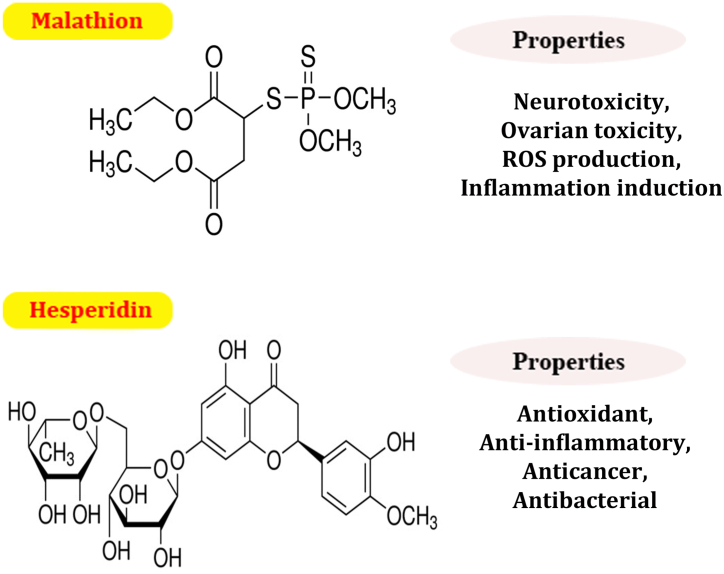
Chemical structures of malathion and hesperidin.

Many toxic substances have the potential to affect the normal functioning of the body's organs. One of these substances, malathion (S-(1,2-dicarbethoxyethyl) O, O-dimethyl dithiophosphate), is widely used in agricultural systems worldwide, especially in developing countries [1]. Malathion (MAL) (Fig. 1), which belongs to the class of organophosphorus compounds, is classified as a pesticide used for both residential pest management and public health applications [1]. As a result, it has the potential to cause harmful effects on the environment and healthy body tissues, and also the use of MLA is banned due to its adverse impacts in many countries [2].

The potential for toxicity, whether subacute, acute, or chronic, may arise from exposure to malathion due to its rapid absorption and metabolism within the body [3]. The potential for toxicity exists in various bodily systems, including but not limited to the liver (which is particularly susceptible), kidney, endocrine system, lung, blood cells, and reproductive systems of both males and females [3]. However, malathion has the potential to affect multiple pathways. It has been reported that the balance between oxidants and antioxidants may be disturbed subsequently to malathion exposure [4]. Therefore, mitochondrial dysfunction and induced cell apoptosis result from a rise in the concentrations of reactive oxygen species (ROS), the primary source of oxidative stress [5]. Additionally, the application of malathion has the capacity to increase the synthesis of pro-inflammatory cytokines, specifically tumor necrosis factor-alpha (TNF-α) and interleukin-1 beta (IL-1β), through the initiation of the nuclear factor-ĸB (NF-ĸB) pathway [6]. This process may result in the occurrence of organ impairment and apoptosis. Multiple studies have demonstrated that exposure to malathion results in elevated levels of malondialdehyde (MDA) within ovarian tissues, leading to apoptosis of granulosa cells and a reduction in the number of healthy follicles [7,8]. Additionally, malathion exposure has been found to decrease the activity of testicular γ-glutamyl transferase and alkaline phosphatase, resulting in damage to seminiferous tubules and abnormal spermatozoa [9]. Malathion has the ability to influence the hypothalamic-pituitary-gonadal (HPG) axis, leading to male and female infertility or infertility [10,11]. This can occur through malathion binding to hormone receptors or the inhibition of hormone secretions, such as LH, FSH, and testosterone. Damage to Leydig/Sertoli cells and granulosa cells in both men and women could contribute to this outcome, respectively [12].

In today's world, nutritional therapy has the potential to reduce malathion-induced toxicity. In this sense, natural compounds such as polyphenols are considered safe, protective, and strong agents against all types of toxicities due to their anti-apoptotic and antioxidant effects. On the other hand, hesperidin (Fig. 1) is a well-known bioflavonoid that can be discovered in citrus fruits, such as lemons and oranges. Numerous studies have shown that this particular bioflavonoid can maintain antioxidant defenses, protect cell function, and maintain organ health by counteracting apoptosis by reducing lipid peroxidation or inflammation by neutralizing inflammatory cytokines [13,14]. Shoorei et al. also showed that adding HES to the culture media of isolated preantral ovarian follicles of mice could improve follicular development in a 3D culture system [15]. The administration of HES could ameliorate testicular damages in different models of injuries induced by toxic agents, such as cisplatin, γ-radiation, and ischemia/reperfusion (I/R) [[16], [17], [18]].

This experimental study was conducted with the aim of investigating the effects of malathion/hesperidin on the ovarian tissue of mice. This research will primarily focus on various aspects such as oxidative stress, hormones, histopathology, and immune reactivity of two protein receptors, namely proliferative cell nuclear antigen (PCNA) and follicle-stimulating hormone receptor (FSHR), which are both involved in maturation and proliferation.

## 2 Materials and methods

### 2.1 Animal Handling and treatment course

This experimental study utilized a sample of 40 balb/c mice of the adult female gender (8 weeks old), with an average weight of 27–30 g. The study adhered to established animal care guidelines, including maintaining appropriate levels of humidity (30%–60 %), temperature (25 °C ± 2 °C), a 12/12 light/dark cycle, and providing ad libitum access to tap water and chow. The experimental procedures of the present research were approved by the Ethics Committee of Birjand University of Medical Sciences (the ethical code is IR.BUMS.REC.1401.104). A group of mice was housed communally for a period of one month, following which they were subjected to random assignment into four distinct groups, comprising.

1) A control group consisting of 10 animals was administered with only normal saline.
2) A group of animals (n = 10) were administered Malathion (Sigma-Aldrich, Germany) via intraperitoneal injection (i.p.) on the right side of the body on a daily basis. The dose administered was 3 mg/kg [19] for a period of 35 days. This group is henceforth referred to as the MAL group.
3) The group administered with malathion and hesperidin (Sigma-Aldrich, Germany) (MAL + HES, n = 10) received simultaneously a daily intraperitoneal injection of 3 mg/kg and 20 mg/kg [20], respectively, for a duration of 35 days.
4) The group consisting of ten subjects was administered solely with hesperidin (HES) on the left side of the body for the identical duration, as previously stated.

Malathion and hesperidin were dissolved in normal saline solution and administered on a daily basis. According to reports, malathion exhibits rapid urinary excretion and possesses a half-life in humans that varies significantly, ranging from 3 to 20 h [21,22]. On the one hand, it has been observed that hesperidin undergoes rapid elimination within the human body, with a half-life of approximately 6 h [23]. On the last day of the experimental period, which was day 35, the animals were anesthetized with a dose of 90/10 mg/kg ketamine/xylazine. Afterward, serum samples were collected for additional hormonal evaluation. In addition, bilateral ovariectomy was performed on each mouse, whereby the right ovaries were exposed to 10 % formaldehyde for 48 h, while the left ovaries were used for biochemical evaluations.

### 2.2 Histopathological assessments

The histopathological evaluation involved embedding the right ovaries in paraffin, followed by serial sectioning at a thickness of 5 μm. The sections were then deparaffinized and stained with hematoxylin and eosin (H&E). Subsequently, the quantities of cystic follicles, as well as primary, secondary, and tertiary follicles, were enumerated according to our prior investigation. In each group, a random selection of 10 out of 15 sections was observed by light microscopy (Nikon, Japan) at a magnification of × 100. In order to prevent duplication of follicle counts, a single follicle was tallied when the presence of a darkly stained nucleolus was observed within its nucleus.

### 2.3 Immunohistochemistry staining

The slides were immersed in a TBS solution (1X, Sigma: T5912) and subjected to microwave irradiation until boiling. Following this, the microwave was turned off, and the samples were allowed to remain in the solution for a duration of 20 min. Subsequently, the specimens underwent a triple wash with phosphate-buffered saline (PBS) (Sigma-P4417) for 5 min. Subsequently, a mixture of H_2_O_2_ (Sigma: 7722-84-1) and methanol at a ratio of 1:9 was prepared, and the slides were subjected to the solution for a duration of 10 min. The specimens underwent PBS washing, followed by incubation with primary antibodies against PCNA and FSHR, which were diluted at a ratio of 1:100 with PBS. The incubation was carried out at room temperature for 1 h. Subsequently, the specimens underwent a triple washing procedure, whereby each wash lasted for a duration of 5 min, and employed PBS. Following this, a volume of 100 μl of the linker (Diagnostics BioSystems-PVP1000D) was introduced to the samples and allowed to incubate for a period of 15 min. Subsequently, the sample underwent three rounds of washing with PBS, followed by the introduction of 100 ml of the linker solution for a duration of 30 min. The specimens were subjected to a washing procedure utilizing PBS followed by the addition of 100 μl of DAB solution (ScyTek-ACV999 The samples were washed with water after 5 min and then placed in hematoxylin dye for 10 s. Next, the slide was subjected to a second round of washing with water. After the subsequent steps of dehydration and transparency, the samples were mounted on the slides and then imaged using a LABOMED optical microscope at a magnification of 400×. Negative control was conducted on four slides using a serial section, wherein FSHR and PCNA antibodies were absent. The immunohistochemistry intensity was evaluated and classified into four grades; absence of staining (0), low intensity (0<low intensity<1), moderate intensity (1< moderate intensity<2), and high intensity (2<heavy intensity<3) [24]. The primary criterion for semi-quantitative evaluation was granulosa cells, and oocytes were used for investigating the IHC intensity of FSHR and PCNA, respectively [25,26].

### 2.4 Antioxidant parameters

In accordance with our earlier research, the evaluation of oxidative stress indices, such as catalase (CAT), superoxide dismutase (SOD), and malondialdehyde (MDA), was performed on the left ovaries of mice. Protein levels in ovarian tissues were calculated using the Bradford technique. After homogenizing samples in 5.1 % potassium chloride solution to obtain 1:10 (w/v) of whole homogenates, lipid peroxidation was assessed using the thiobarbituric acid-reactive species and quantified as MDA levels. Finally, the level of MDA was calculated using a thiobarbituric acid reaction based on a method established by Uchiyama and Mihara [27]. The assessment of SOD activity was conducted through the utilization of commercially available kits (Ransod and Ransel, Randox Com, UK), which were based on the methodology developed by Sun et al. [28]. The CAT activity was assessed through the employment of a commercially available kit (Beyotime Biotechnology, Shanghai, China), wherein the absorbance of the resultant colored product N-(4-antipyrine)-3-chloro-5-sulfonate-*p*-benzoquinoneimine was measured via spectrophotometry at a wavelength of 530 nm [29].

### 2.5 Hormonal assay

The serum samples obtained from the heart blood of mice were evaluated for the levels of hormones, namely progesterone (P4), 17-β estradiol (E2), FSH, and LH. The concentrations of P4 (ng/ml) and E2 (pg/ml) were quantified utilizing suitable laboratory kits. (ELISA Kit, Diaplus, USA, Diagnostic System Laboratories Inc., USA). The amounts of LH and FSH (IU/ml) were measured using ELISA Kits (Sunlong Biotech Co., Ltd.) in accordance with the manufacturer's instructions.

### 2.6 Pro-inflammatory cytokines

The inflammatory parameters, IL-1β and TNF-α, present in the left ovarian tissues were assessed by means of commercially available kits (Sigma Aldrich, Germany) at a wavelength of 450 nm.

### 2.7 Statistical analysis

The statistical analysis of the data was conducted using the SPSS software version 16 (SPSS Inc.). The study employed one-way analysis of variance followed by Tukey's post hoc test to compare the values between different experimental groups. The obtained values were represented as the means and standard deviation (mean ± SD). The level of statistical significance was set at p < 0.05.

## 3 Results

### 3.1 Effects of malathion and hesperidin on body and ovarian weights

Table 1 shows that as compared to the control group, the mean body weight of mice exposed to malathion decreased by 19.49 % (p < 0.05), while the mean body weight of mice exposed to malathion and treated with hesperidin increased by 10 % (p < 0.05). Additionally, no significant (p > 0.05) difference was observed when comparing hesperidin and control groups. Conversely, the average weight of the ovaries in mice that were subjected to malathion exposure exhibited a statistically significant (p < 0.05) reduction in comparison to the control group. The application of hesperidin resulted in a significant (p < 0.05) increase in the average weight of the ovaries in mice treated with malathion, as compared to the mice that were not treated with hesperidin. Regarding this matter, there was no statistically (p > 0.05) significant difference observed between the mice belonging to the control group and those belonging to the group treated with hesperidin.

**Table 1:** effects of *malathion* (MAL) and *hesperidin* (HES) on both body and ovarian weights.

Groups	The weight of body (Mean ± SD)	Ovarian weight (Mean ± SD)
**Control**	31.4 ± 0.025	0.022 ± 0.004
**MAL**	25.28 ± 0.037^a^	0.013 ± 0.004^a^
**MAL + HES**	28.09 ± 0.026 ^ab^	0.018 ± 0.003 ^ab^
**HES**	31.91 ± 0.014 ^bc^	0.024 ± 0.004 ^bc^

SD: standard deviation, (a): shows a statically significant difference compared to the control group; (b) shows a statically significant difference compared to the malathion group; and (c), shows a statically significant difference compared to the malathion + hesperidin group. P < 0.05.

### 3.2 The impact of malathion and hesperidin on ovarian structure

**Fig. 2:**
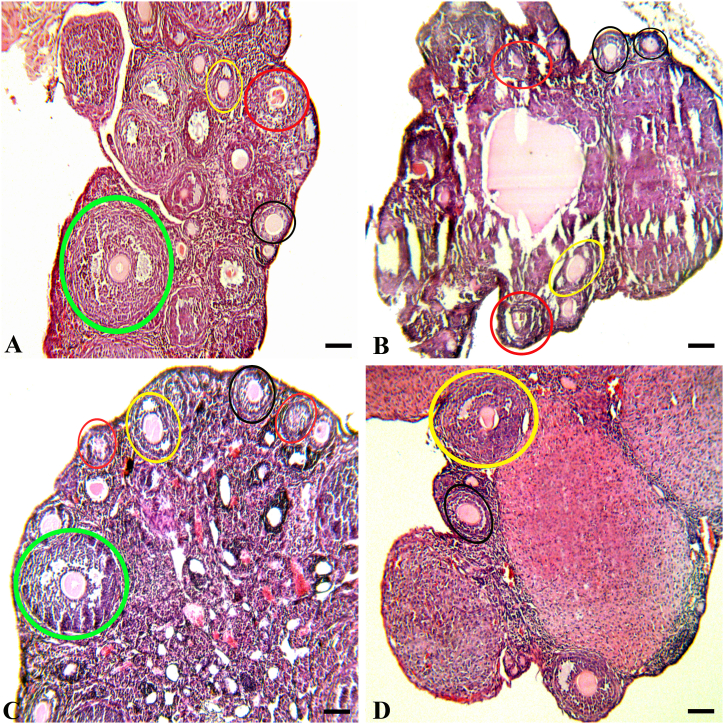
Microscopic photograph of ovarian tissue in all groups of the study (H&E, × 100). (A and D): Control group and Hesperidin group (as the healthy control group), normal ovarian architecture observed in this photograph. (B): Malathion group, abnormal morphological structures, degeneration of stromal interstitial cells, ovarian atrophy, and a significant reduction in the number of follicles observed in this photograph. (C): Malathion + hesperidin group, the sign of atrophy and degeneration of stromal interstitial cells decreased. The arrangement of follicular cells improved. Black, yellow, green, and red circles show primary follicles (when a follicle had a single layer of cuboidal granulosa cells or more than one granulosa cell layer without follicular antrum appearance), secondary follicles (when a follicular antrum appeared within the granulosa layer), tertiary follicles (when a large follicular antrum appeared within the multiple layers of granulosa cells), and atretic follicles (when oocyte degenerated, granulosa cells became loose or eliminated).

Fig. 2 depicts the H&E staining of ovarian tissues. The histopathological findings indicated that the ovarian tissues exhibited typical architecture and morphology, and the follicle count was normal in both the hesperidin-treated and control groups (Fig. 2A, D). The group exposed to malathion exhibited detrimental morphological alterations, such as the degeneration of stromal connective tissue and atrophy in the medullary region. Additionally, there was a higher prevalence of atretic follicles and signs of hemorrhage, as well as loosely arranged follicular cells. (Fig. 2B). The results indicate that the administration of malathion + hesperidin to mice resulted in a reduction in degenerative changes in the medulla of ovaries. Additionally, the observed follicles displayed a normal appearance with the appropriate lining of granulosa cells when compared to the group treated with malathion alone, as depicted in Fig. 2C.

Table 2 displays that in the ovaries of mice treated with malathion, there was a significant (p < 0.05) increase in the number of atretic follicles compared to the control group. In addition, there was a marked (p < 0.05) decrease in the number of follicles in various stages, such as primary, secondary, and tertiary. The administration of hesperidin exhibited a protective effect (p < 0.05) against the toxicity of malathion, thereby preventing the loss of ovarian follicles during the specified stages and reducing the count of atretic follicles. Besides, with regards to the aforementioned variables, there was no statistically (p > 0.05) significant disparity detected upon the comparison of the hesperidin-treated and control groups.

**Table 2:** Ovarian follicle counts in the ovary of mice in all of the studied groups.

Groups	Primary Follicles (Mean ± SD)	Secondary Follicles (Mean ± SD)	Tertiary Follicles (Mean ± SD)	Atretic Follicles (Mean ± SD)
**Control**	12.04 ± 2.21	7.32 ± 0.91	3.03 ± 0.83	3.12 ± 0.26
**MAL**	7.11 ± 1.45^a^	2.35 ± 0.86^a^	1.02 ± 0.91^a^	8.11 ± 1.03^a^
**MAL + HES**	9.38 ± 1.93 ^ab^	4.17 ± 0.93 ^ab^	2.25 ± 0.58 ^ab^	5.17 ± 0.64 ^ab^
**HES**	12.2 ± 2.42 ^bc^	7.81 ± 1.01 ^bc^	3.27 ± 0.69 ^bc^	3.08 ± 0.29 ^bc^

The number of ovarian follicles of mice in control, malathion (3 mg/kg, i.p. injection, daily, for 35 days), malathion + hesperidin, and hesperidin (20 mg/kg, i.p. injection, daily, for 35 days) groups. SD: standard deviation. (a): shows a statically significant difference compared to the control group; (b) shows a statically significant difference compared to the malathion group; and (c), shows a statically significant difference compared to the malathion + hesperidin group. P < 0.05.

### 3.3 Malathion and hesperidin effects on the immunoreactivity of PCNA and FSHR proteins

**Fig. 3:**
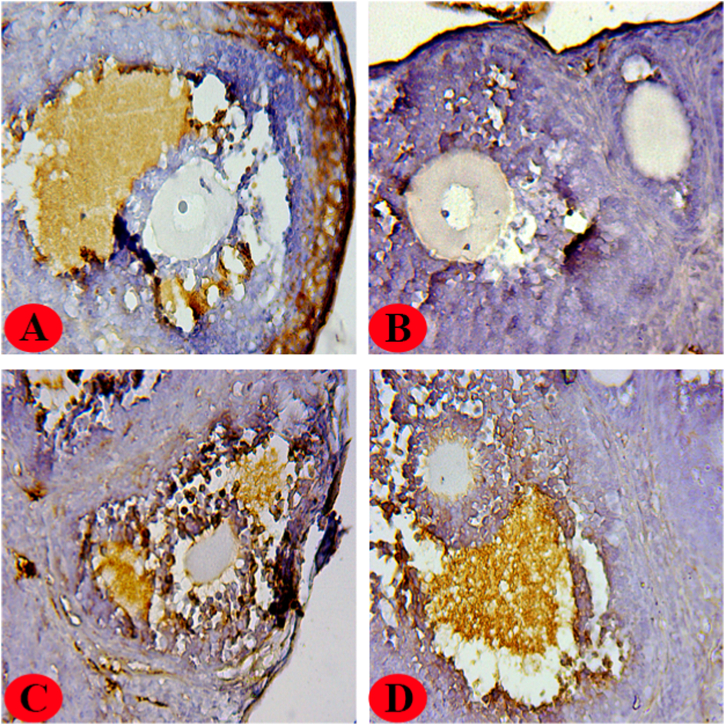
Immunoreactivity of FSHR in ovaries of all groups of the study. (A): Control group, heavy FSHR staining was detected in granulosa cells. (B): Malathion group, the density of immunohistochemical FSHR staining was low (intensity<1, Table3). (C): Malathion + Hesperidin group, low to moderate FSHR staining (1<intensity<2, Table3) was detected. (D): Hesperidin group, heavy FSHR staining was detected. Thickness of sections (4–5 μm), magnification (x40).

**Fig. 4:**
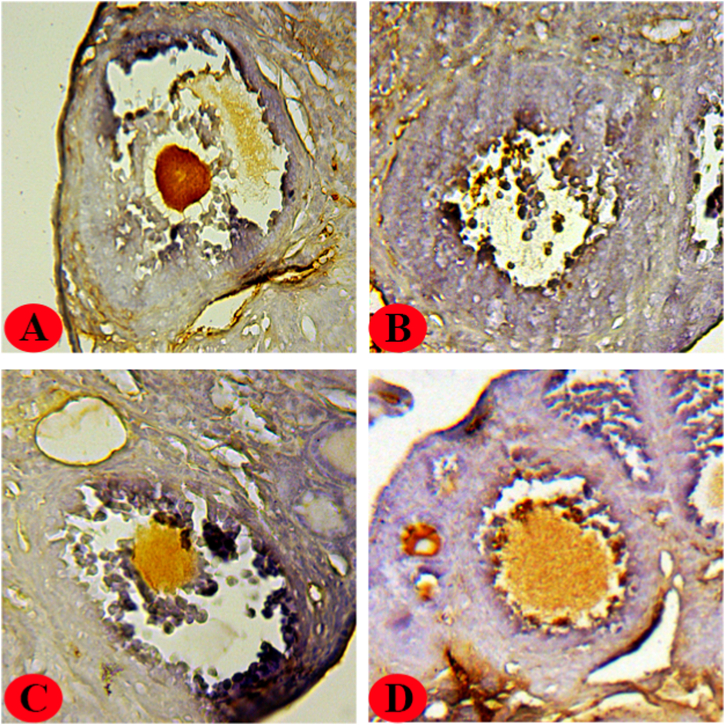
Immunoreactivity of PCNA in ovaries of all groups of the study. (A): Control group, heavy PCNA staining was detected in oocyte. (B): Malathion group, the density of immunohistochemical PCNA staining was low (intensity<1, Table3). (C): Malathion + Hesperidin group, low to moderate PCNA staining (1<intensity<2, Table3) was detected. (D): Hesperidin group, heavy PCNA staining was detected. Thickness of sections (4–5 μm), magnification (x40).

FSHR (Fig. 3) and PCNA (Fig. 4) antibodies were used to evaluate the effects of malathion and hesperidin on ovarian tissues in all groups. These antibodies are thought to be indicators of proliferation and maturation. The intensity of immunohistochemical staining results is also presented in Table 3.

**Table 3:** Grading immunoreactivity of proliferating cell nuclear antigen (PCNA) and follicle-stimulating hormone receptor (FSHR) in all groups of the study.

Groups	FSHR (Mean ± SD)	PCNA (Mean ± SD)
**Control**	2.88 ± 0.47	2.67 ± 0.04
**MAL**	0.88 ± 0.01^a^	0.35 ± 0.04^a^
**MAL + HES**	1.95 ± 0.32 ^ab^	1.65 ± 0.03 ^ab^
**HES**	2.96 ± 0.12 ^bc^	2.73 ± 0.04 ^bc^

SD: standard deviation, (a): shows a statically significant difference compared to the control group; (b) shows a statically significant difference compared to the malathion group; and (c), shows a statically significant difference compared to the malathion + hesperidin group. P < 0.05.

The present study demonstrates that the immunoreactivity of PCNA and FSHR was predominantly observed in oocytes and granulosa cells, respectively, as depicted in Fig. 3, Fig. 4. The malathion-treated group exhibited minimal to negligible staining intensity in the ovaries for the two antibodies. Also, the malathion + hesperidin group showed minimal to moderate immunoreactivity against PCNA and FSHR. Furthermore, it was observed that in both control groups (control and hesperidin-treated), there was an obvious presence of PCNA and FSHR staining in oocytes and granulosa cells, respectively.

### 3.4 The impact of malathion and hesperidin on antioxidant status

**Fig. 5:**
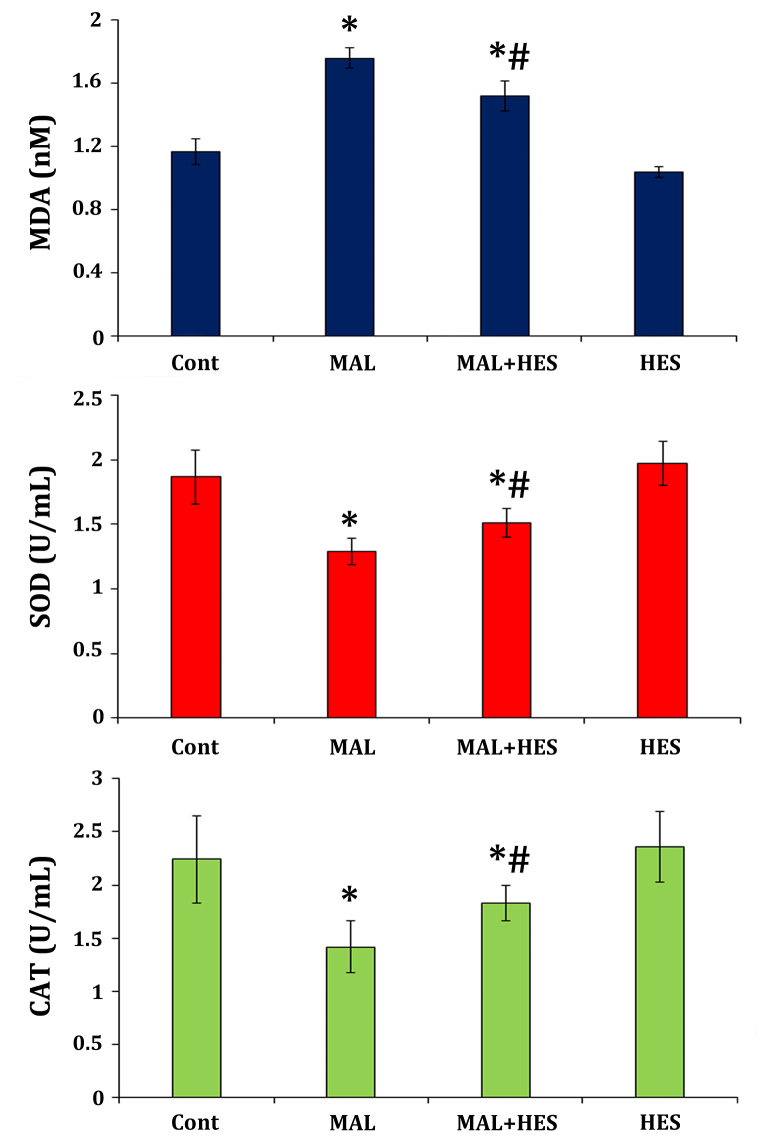
The effect of malathion (MAL) and hesperidin (HES) on the tissue levels of antioxidant factors in the ovaries of mice. Exposure to malathion increased MDA levels and decreased the activity of SOD and CAT compared to the control group (p < 0.05), while treatment with hesperidin there is a significant improvement (p < 0.05) compared to the MAL-untreated group. (*): shows a statically significant difference compared to the control group; (#) shows a statically significant difference compared to the malathion group. MDA (malondialdehyde), SOD (superoxide dismutase), and CAT (catalase).

The effectiveness of hesperidin in mitigating the impact of malathion was assessed by analyzing the concentrations of SOD, CAT, and MDA in the left ovarian tissues of mice across all groups. The findings depicted in Fig. 5 indicate that the concentration of MDA in the ovarian tissues of mice that were subjected to malathion exposure demonstrated a statistically (p < 0.05) significant increase in comparison to the control group. Additionally, mice treated with malathion + hesperidin had lower MDA levels than mice treated with malathion alone (p < 0.05). Contrarily, malathion reduced the activity of two antioxidant enzymes, CAT and SOD, in comparison to the malathion-treated group compared with the control group (p < 0.05).

The present study observed a significant (p < 0.05) increase in the activity of SOD and CAT antioxidants in ovarian tissues of the malathion + hesperidin group compared to the malathion-treated group. Furthermore, the levels of CAT, SOD, and MDA levels did not exhibit a significant (p > 0.05) alteration when a comparative analysis was performed between hesperidin-treated and control groups.

### 3.5 The role of hesperidin and malathion on hormonal levels

The comparison of serum levels of various hormones, including P4, E2, FSH, and LH, in all research groups, is shown in Table 4. All of the above hormone levels were considerably (p < 0.05) lower in mice in the malathion-treated group compared with the control group. Additionally, a significant (p < 0.05) rise in all of the above-mentioned hormones was detected in mice receiving both malathion and hesperidin in comparison to the malathion-treated group. Furthermore, there was no significant (p > 0.05) difference in the levels of P4, E2, FSH, and LH when a comparison was made between two control and hesperidin-treated groups.

**Table 4:** Serum levels of hormones in mice in all of the studied groups.

Groups	E2 (ng/mL) (Mean ± SD)	P4 (ng/mL) (Mean ± SD)	LH (mIU/mL) (Mean ± SD)	FSH (mIU/mL) (Mean ± SD)
**Control**	20.45 ± 1.14	1.96 ± 0.01	7.67 ± 0.28	11.21 ± 0.17
**MAL**	13.66 ± 1.21^a^	1.08 ± 0.01^a^	3.56 ± 0.51^a^	6.17 ± 0.93^a^
**MAL + HES**	17.38 ± 1.57 ^ab^	1.41 ± 0.01 ^ab^	5.83 ± 0.51 ^ab^	8.93 ± 0.71 ^ab^
**HES**	21.21 ± 1.37 ^bc^	1.96 ± 0.01 ^bc^	7.86 ± 0.43 ^bc^	11.68 ± 0.34 ^bc^

SD: standard deviation. E2 (17β-estradiol), P4 (progesterone), LH (luteinizing hormone), FSH (follicle‐stimulating hormone). (a): shows a statically significant difference compared to the control group; (b) shows a statically significant difference compared to the malathion group; and (c), shows a statically significant difference compared to the malathion + hesperidin group. P < 0.05.

### 3.6 The impact of hesperidin and malathion on pro-inflammatory mediators

Previous research has indicated that the expression of IL-1β and TNF-α in different follicular compartments is important for follicular development, oocyte maturation, and regulation of ovarian steroid synthesis [30,31]. The present study aimed to investigate the effect of malathion and hesperidin on ovarian physiology by analyzing the levels of these pro-inflammatory cytokines in ovarian tissues.

The results depicted in Table 5 indicate a significant (p < 0.05) elevation in the levels of TNF-α and IL-1β in the ovary of mice that were subjected to malathion exposure, as compared to the control group. The levels of cytokines were significantly reduced in the malathion + hesperidin group compared to the malathion-treated mice (p < 0.05). However, there was no statistically significant difference (p > 0.05) between the control and HES-treated groups when the levels of the aforementioned cytokines were compared (Table 5).

**Table 5:** Tissue concentrations of proinflammatory cytokines, namely TNF-α and IL-1β, in the ovary of mice.

Groups	TNF-α (Mean ± SD) (pg/mg protein)	IL-1β (Mean ± SD) (pg/mg protein)
**Control**	27.91 ± 3.55	10.38 ± 2.11
**MAL**	65.14 ± 7.23^a^	21.14 ± 4.24^a^
**MAL + HES**	48.12 ± 4.11 ^ab^	15.32 ± 4.07 ^ab^
**HES**	25.32 ± 4.62 ^bc^	10.17 ± 2.56 ^bc^

SD: standard deviation, (a): shows a statically significant difference compared to the control group; (b) shows a statically significant difference compared to the malathion group; and (c), shows a statically significant difference compared to the malathion + hesperidin group. P < 0.05.

## 4 Discussion

Malathion and other organophosphorus compounds are widely used as pesticides in agricultural practices worldwide. However, prolonged exposure to these chemicals may have adverse effects on fertility. Although malathion exposure has been linked to health-related complications, such as loss of ovarian follicles, irregular menstrual cycles, and disturbance in the HPG axis, the precise mechanism by which malathion affects the female reproductive system is still opaque.

The present study aimed to examine the effects of MAL on the expression of proteins associated with proliferation and maturation, namely PCNA and FSHR, through an experimental approach. We investigated the biochemical, histopathological, and hormonal alterations resulting from malathion toxicity, as well as the potential mitigating effects of hesperidin treatment on these parameters.

Our findings indicated that malathion exposure in mice resulted in a significant reduction in ovarian and total body weight as compared to the control group. However, the administration of hesperidin was found to effectively mitigate the negative impacts of malathion on both these parameters.

According to reports, when mice were administered malathion daily for a period of 15 days via subcutaneous injection at doses of 30 and 100 mg/kg, there was no observed impact on their body weight [32]. A different study documented that prepubertal male mice, following a one-month oral exposure to malathion at a dose of 200 mg/kg, exhibited a decrease in body weight and an increase in the proportional weights of the liver and kidney [33]. Additionally, a 2-month period of oral administration of malathion (0–108 mg/kg) for male Wistar rats resulted in dose-dependent reductions in testicular and body weights [34]. likewise, when exposed to malathion (0.001 mg/l), the body and ovarian weights of the animals were significantly diminished [35]. In contrast, female rats administered varying doses (50–150 mg/kg) of diazinon, an organophosphorus compound, orally for a duration of 2 weeks did not exhibit any significant alterations in body weight. However, a dose-dependent reduction in ovarian weight was observed [36]. Hence, there is controversy regarding the effect of malathion on body functions and organ weight. On the one hand, one study reported that treatment with 100 mg/kg of HES (orally and daily for 8 days) increased the ovarian weight of rats in a model of ovarian toxicity induced by cyclophosphamide [37].

During the second stage of our study, a histopathological evaluation was conducted using H&E staining. The results indicated notable damage to the ovaries of female mice that were exposed to malathion for a duration of 35 days. The use of malathion was found to impede the typical physiological functioning of the ovaries by reducing the count of viable follicles, encompassing primary, secondary, and tertiary follicles, and causing atresia in the ovarian tissue. The findings of the study indicate that the administration of hesperidin resulted in a reduction in the count of atretic follicles and an improvement in the morphology and architecture of ovaries in mice treated with malathion.

Consistent with our findings, Ozsoy and colleagues revealed that oral administration of malathion exposure at a dose of 100 mg/kg resulted in damage to ovarian tissue, including hemorrhage, vascular congestion, inflammatory cell infiltration, follicular degeneration, and edema [38]. However, they also observed that treatment with lipid emulsion at a dose of 3 ml/kg via intravenous injection was able to mitigate the toxic effects of malathion on the ovaries. Oral treatment of diazinon (50–150 mg/kg for two weeks) had no influence on the number of ovarian follicles at different stages, according to another study examining the effects of diazinon on ovarian morphology [36]. Besides, ovarian tissues from rats given malathion 33 mg/kg (i.p.) for 15 days showed an increase in atretic follicles and a decrease in the number of normal follicles [39]. Moreover, it has been reported that HES was able to decrease ovarian oxidative damage induced by aluminium phosphide through the activation of glutathione peroxidase (GPx) and SOD [37]. Also, Abolaji et al. reported that HES (100 and 200 mg/kg/day, orally) administration for a 4-week duration preserved the histological structure of the ovary of rats exposed to the ovotoxicity of 4-vinylcyclohexene diepoxide (VCD) [40].

However, it has been hypothesized that follicular atresia is associated with apoptosis of the ovarian granulosa cells [41]. Multiple experimental studies that examined the effects of pesticide exposure at nanomolar concentrations reached comparable results on granulosa cell death [8,42]. It has been observed that the daily treatment of mice with malathion at a dose of 30 mg/kg for a period of 35 days could result in the elevation of free radical agents in the ovarian tissue, promoting the autophagic and apoptotic pathways. This phenomenon is molecularly corroborated by the presence of the decreased expression levels of sequestosome 1 (P62) and Bcl-2, as well as the increased expression rates of light chain 3B (LC3B) and caspase-3 [19]. Therefore, we conclude that the activation of autophagy and apoptosis in granulosa cells of the ovarian tissue is responsible for both the rise in atretic follicles and the deterioration in ovarian function (here, defined as the number of follicles at various stages). In the final stage, we found that the ovarian tissues of malathion-exposed mice showed variations in the concentrations of antioxidant indices, such as CAT, SOD, and MDA. Hesperidin, however, has the potential to boost CAT and SOD activity while decreasing MDA production as a result of its ROS-scavenging effects.

The etiology of ovarian pathology may be attributed, at least in part, to the oxidative stress that is triggered by exposure to organophosphate chemicals. Malathion has the ability to modify the cellular antioxidant system by increasing lipid membrane peroxidation, which can lead to excessive production of MDA. The level of MDA is commonly utilized as an indicator of oxidative stress [1]. Several lines of evidence have shown that malathion exposure can impair the activity of natural antioxidant defense enzymes, including GPx and SOD, in various tissues, leading to a rise in MDA levels [38,43,44]. Two weeks of malathion (50 mg/kg) exposure dramatically increased MDA synthesis in the ovaries of rats, as reported by Arab et al. [7]. The excessive production of MDA as a result of malathion exposure can lead to the activation of genes associated with apoptosis, including p53 and caspase-3/9. This can result in the fragmentation of nuclear DNA and, ultimately, cell death. Several studies have reported that the administration of hesperidin can lower the generation of ROS and MDA (oxidative stress reduction), as well as increase the levels of endogenous antioxidant defense enzymes in ovarian tissues [37,45,46]. The enzymatic antioxidant defenses, namely CAT and SOD, are responsible for the conversion of active oxygen molecules into non-toxic compounds. This process involves the conversion of O_2_−. to H_2_O_2_ and subsequently to H_2_O and O_2_ [47]. The enzyme SOD is present in all living organisms and plays a crucial role in safeguarding aerobic cells from oxidative stress [48]. On the other hand, CAT is a heme protein that exists in a tetrameric form and exhibits alternative divalent oxidation and reduction at its active site when exposed to H_2_O_2_ [49]. The findings indicate that hesperidin possesses notable chelating and antioxidant properties, which effectively mitigate oxidative stress, resulting in a decrease in the restoration of normal physiological activity and pathological alterations. During the fourth stage of the experiment, a decrease in the immunoreactivity of antibodies against FSHR and PCNA was observed in the ovaries of the-malathion treated group. In addition, in the group treated with both malathion and hesperidin, a significant increase in the immunoreactivity of these antibodies was detected.

The FSH receptor is classified as a G protein-coupled receptor and is mainly found in the granulosa cells of mature ovarian follicles [50]. Through intricate biological pathways, FSH and its cognate receptor can combine to stimulate cAMP synthesis in granulosa cells [51]. Additional functions of cAMP include the initiation of terminal differentiation (maturation), the stimulation of granulosa cell proliferation, and the inhibition of follicle expansion [52]. This suggests that FSHR is a target involved in proliferation and maturation. Our data showed that in malathion-treated mice, FSHR expression and immunoreactivity were both low, whereas, in the malathion + hesperidin group, both increased to near-moderate levels. In the mouse model of cyclophosphamide-induced premature ovarian failure, we found that FSHR mRNA expression was significantly decreased [53]. A study conducted by Liu et al. revealed a significant decrease in the count of murine ovarian granulosa cells expressing FSHR when treated with ethanol [54]. An additional finding from our study indicated that the level of PCNA immunoreactivity was significantly low (almost negligible) in the ovaries of mice treated with malathion. However, in the group of mice that were administered both malathion and hesperidin, the expression of PCNA was moderately observed. PCNA is a non-histone protein with a molecular weight of 36 kDa that serves as an auxiliary component of DNA polymerase-δ enzymes, playing a crucial role in DNA synthesis. It is widely utilized as a standard marker in cells undergoing proliferation [55]. The involvement of PCNA in cell survival, DNA repair, and cell cycle control has been demonstrated [56,57]. According to reports, PCNA expression has been detected in the ovaries of both adult and newborn mammals [58,59]. The ovarian follicle development process is significantly influenced by a crucial regulator. This regulator is also associated with mitotic activity, making it a potential target for both maturation and proliferation [60]. Furthermore, it has been observed that the expression of PCNA is prominent in oocytes during the follicular growth phase, while its expression is comparatively lower in somatic cells such as theca and granulosa cells within the ovarian tissue [25,61]. In a study carried out by Gouveia et al., it was observed that the administration of cisplatin had the potential to decrease the proportion of cells expressing PCNA in murine ovarian follicles. Also, pretreatment with N-acetylcysteine, which acted as an antioxidant source, was found to enhance the immunoreactivity of PCNA [62]. Furthermore, in our previous research, we found that coenzyme Q10 treatment increased PCNA mRNA expression in cyclophosphamide-treated mouse ovaries [53]. A study performed by Sargazi et al. demonstrated a statistically significant decrease in the mean number of cells expressing PCNA in the ovaries of rats treated with diazinon, an organophosphorus pesticide administered at a dosage of 60 mg/kg intraperitoneally for a period of two weeks. However, the co-administration of vitamin E was found to increase the expression of PCNA [63]. Therefore, it can be inferred that the ovarian toxicity induced by malathion may be associated with alterations in the expression of FSHR and PCNA. It is strongly recommended that future studies employ FSHR/PCNA knockdown mice to elucidate the precise mechanism by which malathion affects the expression of the aforementioned proteins.

The fifth phase of our ongoing investigation examines the effects of malathion and hesperidin on a range of hormones, such as LH, FSH, P4, and E2. The administration of Malathion led to a noteworthy decrease in the serum levels of ovarian hormones (P4 and E2) and reproductive pituitary hormones (FSH and LH). However, the administration of hesperidin was found to be effective in the restoration of these hormone levels.

The precise pathways by which malathion treatment affects reproductive hormone levels in female mice are not yet fully understood. However, existing research has demonstrated that exposure to environmental toxins, including various organophosphorus pesticides, can diminish steroidogenesis in both male and female subjects, as well as lower levels of FSH and LH [9,64,65]. The literature has documented that the organophosphorus category of pesticides has the potential to elicit endocrine disruption, specifically at the hypothalamic-pituitary-gonadal (HPG) axis [66]. The potential impact of hesperidin administration on serum levels of LH, FSH, progesterone, and estrogen has been reported in the literature [67,68]. Elnahas et al. showed that administration of HES (80 mg/kg/day, i.p) increased serum levels of E2 and FHS in a premature ovarian failure model induced by cyclophosphamide in rats [69]. It is suggested that this effect may be attributed to the antioxidant and anti-inflammatory properties of hesperidin. The observed decrease in plasma levels of E2 and P4 may be ascribed to the suppressive effect exerted by malathion on the excretion of LH and FSH from the anterior pituitary gland [12]. The reproductive endocrine axis has a regulatory role in the growth of follicles. Therefore, it can be assumed that the reduction of FSH levels in receptor expression inhibits follicular growth, thus preventing the increase of serum concentrations of P4 and E2. Therefore, it is highly recommended that forthcoming research endeavors aim to determine the precise effects of MAL on ovarian function and hormonal production by examining the expression of crucial genes implicated in steroidogenesis, including HSD17β, HSD3β, and StAR, along with Kisspeptin genes that are predominantly situated in the hypothalamus and play a pivotal role in regulating the reproductive cycle of hormones by exerting influence on the HPG axis. Finally, in the sixth stage, our results showed that exposure to malathion significantly increased IL-1β and TNF-α levels in murine ovaries, while hesperidin injection decreased these levels.

Malathion has been linked in multiple studies to the initiation of the production of inflammatory cytokines in a variety of cell types [[70], [71], [72]]. An experiment was conducted in which male rats were orally administered various doses of malathion for 30 days. The results showed a significant increase in mRNA expression of pro-inflammatory cytokines, namely IL-1β and TNF-α, in the liver tissue of rats treated with malathion (50–200 mg/kg) compared to the control group. However, taurine administration was found to reduce the deleterious effects of malathion on the cytokines of the previously mentioned factors [73]. In addition, the inhibitory effects of hesperidin on the NF-κB pathway have been hypothesized to be responsible for the anti-inflammatory effects of this compound [74]. Nevertheless, ovarian macrophage-secreted cytokines such as interleukins and tumor necrosis factors can modulate ovarian cell responsiveness to pituitary gonadotropins [75]. Ovarian steroidogenesis can be suppressed, and follicular atresia can be induced if ovarian granulosa cells produce too much TNF-α via activating the NF–B signaling pathway [76,77]. The components of the IL-1 system, e.g., IL-1β, have been identified in oocytes and granulosa/theca cells. Despite a limited understanding of the precise role of the IL-1 family, excessive expression of IL-1 has been observed to impede the formation of LH and FSH receptors, as well as influence the secretion of both P4 and E2 [75,78]. In our investigation, low ovarian (P4 and E2) and pituitary (FSH and LH) hormone serum concentrations, FSHR expression, as well as the abundance of atretic follicles were all associated with elevated levels of the above-mentioned pro-inflammatory cytokines. To further clarify the effects of malathion on inflammation induction, we recommend that the NF-κB pathway be studied in subsequent research.

## 5 Conclusion

The findings of our study demonstrate that malathion has the potential to cause ovarian dysfunction by downregulating the expression of FSHR and PCNA, which play crucial roles in the maturation and proliferation of oocytes and granulosa cells, respectively. This is the first time that such an effect of malathion on ovarian function has been reported. Administration of malathion leads to a decrease in the number of ovarian follicles, a change in the function of the HPG axis with a significant decrease in the production of hormones, such as P4, E2, FSH, and LH, along with an increase in the count of atretic follicles and the production of pro-inflammatory cytokines (TNF-α and IL-1β). The use of hesperidin as an antioxidant agent showed a satisfactory effect in regulating and reducing the adverse consequences of malathion on the functionality of ovaries. It is strongly recommended that forthcoming studies employ FSHR/PCNA knockdown mice to assess the precise effects of malathion on ovarian dysfunction. Additionally, these studies should explore inflammation pathways such as NF-κB, as well as genes implicated in the HPG axis and steroidogenesis process. However, since this study had financial limitations, we strongly suggest that other scientists to better understand the positive properties of hesperidin in ameliorating adverse effects of toxic agents such as malathion should examine other doses of hesperidin to find out the optimum dose.

## Data Availability statement

It would be available via reasonably requests.

## CRediT authorship contribution statement

**Mahnaz Zarein:** Project administration, Data curation, Conceptualization. **Asghar Zarban:** Writing – review & editing. **Hamed Shoorei:** Supervision, Writing – review & editing. **Mehdi Gharekhani:** Software. **Mohammadmehdi Hassanzadeh-Taheri:** Methodology, Writing – original draft.

## Declaration of competing interest

The authors declare that they have no known competing financial interests or personal relationships that could have appeared to influence the work reported in this paper.
